# Effects of *Chimonanthus nitens* Oliv. Leaf Extract on Glycolipid Metabolism and Antioxidant Capacity in Diabetic Model Mice

**DOI:** 10.1155/2017/7648505

**Published:** 2017-09-19

**Authors:** Hui Chen, Yan Jiang, Zhanwei Yang, Wenbing Hu, Lei Xiong, Ning Wang, Xin Liu, Guodong Zheng, Kehui Ouyang, Wenjun Wang

**Affiliations:** ^1^Key Lab for Agro-product Processing and Quality Control of Nanchang City, College of Food Science and Engineering, Jiangxi Agricultural University, Nanchang 330045, China; ^2^College of Animal Science and Technology, Jiangxi Agricultural University, Nanchang 330045, China

## Abstract

The paper investigated the antihyperglycemic and antihyperlipidemic efficacy and antioxidant capacity of *Chimonanthus nitens* Oliv. leaf extract (COE) in combination of high-glucose-fat diet-fed and streptozotocin-induced diabetic model mice. Various physiological indexes in diabetic model mice were well improved especially by oral administration of high dose of COE; the results were listed as follows. Fast blood glucose (FBG) level and serum triglyceride (TC), total cholesterol (TG), low-density lipoprotein cholesterol (LDLC), and malondialdehyde (MDA) as well as MDA in liver were significantly reduced; fasting serum insulin (FINS) and insulin sensitivity index (ISI) were both increased; high-density lipoprotein cholesterol (HDLC) in serum was significantly increased; total antioxidant capacity (T-AOC), activities of superoxide dismutase (SOD), glutathione peroxidase (GSH-Px), and catalase (CAT) in serum and liver were apparently enhanced; liver coefficient (LC), liver transaminase, and alkaline phosphatase (ALP) were decreased. Furthermore, pancreas islets and liver in diabetic model mice showed some extend of improvement in morphology and function after 4 weeks of COE treatment. In consequence, COE was advantageous to regulate glycolipid metabolism and elevate antioxidant capacity in diabetic model mice. Thus, the present study will provide a scientific evidence for the use of COE in the management of diabetes and its related complications.

## 1. Introduction

Diabetes mellitus (DM) is one of the high incidences of metabolic disorder syndromes which was characterized by high blood glucose level. It is of genetic susceptibility, related with the environmental factors, and mainly caused by insulin secretion or insulin action disorder [1]. So far, there have been several types of DM found; type two diabetes mellitus (T2DM) accounts for approximately 90% in diabetes prevalence [2, 3]. Chronic hyperglycemia is a key factor caused diabetic complications, such as cardiovascular disease, neurodegenerative disease, and retinopathy [4–8]. Therefore, it is important for diabetics to control blood glucose level in normal. Chronic hyperglycemia also termed glucose toxicity has a strong impact of damage on beta-islet cell failure which origins from high glucose-inducing superfluous reactive oxygen species to aggravate oxidative stress in body tissue [9, 10]. There are also multiple abnormalities of lipoprotein metabolism due to chronic hyperglycemia, including elevated levels of total cholesterol (TC) and triglycerides (TG) in serum in diabetes [11]. Liu et al. [12] found that the improvement on glycolipid metabolism was related with the level of oxidative stress in type 2 diabetic model mice. On the whole, glucose, lipid metabolism, and oxidative stress have interaction with each other in diabetes.

*Chimonanthus nitens* Oliv. leaf is also called Yan Ma Sang, Xiang Feng tea, or Mao Shan tea and deemed to be a very important Chinese traditional medicine. Chen and Liu [13] have reported that *Chimonanthus nitens* Oliv. leaves had the function of reducing weight, antiappetite, and reducing TG and TC levels in obese model mice. Another study demonstrated once again that *Chimonanthus nitens* Oliv. leaf alcohol extract affected the synthesis of body fat and decreased fat index, TG, and TC obviously; moreover, the extract had no significant toxic effects to the mice [14]. In addition, it was reported that *Chimonanthus nitens* Oliv. leaf alcohol extract had favourable antioxidant ability *in vitro* [15]. Our previous study [16] had reported that ethanol extracts of *Chimonanthus nitens* Oliv. leaves had inhibitory effect on *α*-glucosidase activity *in vitro* in different degrees, and the fraction of 50% ethanol eluate (EE) showed the highest effect on *α*-glucosidase activity, which suggested *Chimonanthus nitens* Oliv. leaves possessed the potential of the hypoglycemic activity. In this study, we would further explore whether *Chimonanthus nitens* Oliv. leaves could exercise effects of antihyperglycemic, antihyperlipidemic, and antioxidant capacity on the diabetic model mice induced by the combination of high-glucose-fat diet-fed and intraperitoneal injection of streptozotocin.

## 2. Materials and Methods

### 2.1. Materials and Reagents

The dried *Chimonanthus nitens* Oliv. leaves were purchased from a local merchant in Sanqing Mountain of Yushan County (Jiangxi, China). Streptozotocin (STZ) was purchased from Sigma Chemical Co. (MO, USA). Hematoxylin and eosin dye solution was purchased from Beijing Jiuzhou Berlin Bio-Technology Co. Ltd (Beijing, China). All the other chemicals and solvents were analytical grade unless otherwise stated.

### 2.2. The Preparation of *Chimonanthus nitens* Oliv. Leaf Extract (COE)

*Chimonanthus nitens* Oliv. leaves were processed as the methods described in our previous study [16]. The fraction of 50% ethanol eluate which exhibited the notable inhibition with IC_50_ of 0.376 mg/mL among all ethanol eluates was selected and applied to our present study. The fraction of 50% ethanol eluate was defined as *Chimonanthus nitens* Oliv. leaf extract mentioned in this paper.

### 2.3. Animal and Induction of Diabetes

All animal experiments were conducted following the Guide for the Care and Use of Laboratory Animals of the National Institutes of Health. Kunming (KM) male mice (23–27 g, lot number 43004700027472, purchased from Hunan SJA Laboratory Animal Co. Ltd, Hunan, China) were kept in polycarbonate cages at the university animal house under controlled conditions (temperature: 18–22°C, humidity: 55–60%, light: 12 h light/12 h dark cycle).

After all mice adjust to the new surroundings for three days with free access to food and water, they were allocated with two dietary regimens. Eight KM male mice selected randomly from them were fed with normal diet (14.2% fat, 18.5% protein, and 59% carbohydrates, as a percentage of total kcal), and the remaining 32 KM male mice were fed with high-glucose-fat diet (18% lard oil, 20% sucrose, and 3% egg yolk powder, 59% normal diet, as a percentage of total weight) for an initial period of 4 weeks. All of them received water *ad libitum*. High-glucose-fat diet-fed mice were applied to induce hyperglycemia by intraperitoneal injection of STZ (80 mg/kg body weight) in 0.1 M freshly citrate buffer (pH 4.5) after fasting for 12 h once a day for two successive days. Normal diet-fed mice were injected with an equal volume of 0.1 M citrate buffer. Mice with fasting blood glucose being more than 11.1 mmol/L and kept steady for three days were considered as diabetic. Blood samples were collected from caudal vein for determining blood glucose level.

### 2.4. Experimental Design

The 32 diabetic model mice were divided randomly into 4 groups on average listed in Table 1, including diabetic model group (DM), low dose of *Chimonanthus nitens* Oliv. leaf extract group (COE-L), high dose of *Chimonanthus nitens* Oliv. leaf extract group (COE-H), and rosiglitazone group (ROS). Eight healthy mice were assigned to the normal control group (NC). The treatment corresponding to each group was described in Table 1 and performed at 8:00 am to 9:00 am (Beijing time) every day. After 4 weeks of treatment, the animals were fasting for 12 h then anesthetized and sacrificed for the experiment. Blood samples were collected from the heart, poured into blood-collecting tube, and then centrifuged (3000 r/min) at 4°C for 10 min to obtain serum which was used for biochemical studies. Pancreas and liver were carefully removed, rinsed by 0.9% sodium chloride solution, and dried by filter paper. Then, pancreas was fixed in PBS containing 10% formalin. Liver was weighed to calculate liver coefficient (LC) according to the equation as follows: LC = liver mass × 1000/body weight. Next, liver was dissected into two pieces, one was fixed in PBS containing 10% formalin, and another was stored in −80°C until analyzed.

### 2.5. Determination of Fasting Blood Glucose (FBG) Level

FBG was measured with a glucometer (Johnson Medical Equipment Co. Ltd, Shanghai, China) after fasting for 8 h.

### 2.6. Oral Glucose Tolerance Test (OGTT)

The OGTT [17] was performed after 3 weeks of treatment, which was determined in response to the oral administration of glucose (2.0 g/kg body weight) after fasting for 8 h. Blood was collected from the tail veins of all mice to be used for measuring blood glucose level at 0 h, 0.5 h, 1 h, and 2 h after intragastric administration of glucose. Area under the curve (AUC) was calculated according to the computation formula of approximate trapezoidal area as follows:

(1) $$\begin{array}{c} AUC=\left(A+B\right)\times 0.5÷2+\left(B+C\right)\times 0.5÷2+\left(C+D\right)\times 1÷2, \end{array}$$

where *A*, *B*, *C*, and *D* were corresponding to the blood glucose levels of time at 0 h, 0.5 h, 1 h and 2 h, respectively.

### 2.7. Serum Parameter Assay and Liver Antioxidant Activity Determination

The levels of fast serum glucose (FSG), insulin (FINS), and serum lipids including total cholesterol (TC), triglyceride (TG), high-density lipoprotein cholesterol (HDLC), and low-density lipoprotein cholesterol (LDLC) and liver function indexes including serum glutamic-pyruvic transaminase (ALT), aspartate aminotransferase (AST), and alkaline phosphatase (ALP) were quantified separately with commercial kits (Beijing Leadman Biochemical Co. Ltd, Beijing, China) and analyzed by the Beckman coulter AU680 automatic biochemical analyzer (American Beckman Coulter Co. Ltd). Content of malondialdehyde (MDA), total antioxidant capacity (T-AOC), activity of superoxide dismutase (SOD), glutathione peroxidase (GSH-Px), and catalase (CAT) in serum and liver were analyzed with commercial kits from Nanjing Jiancheng Bioengineering Institute (Jiangsu, China) according to the manufacturer's protocols. Insulin sensitivity index (ISI) was calculated by the following equation:

(2) $$\begin{array}{c} ISI=-\text{ln}\left(FSG\times FINS\right), \end{array}$$

where FSG was fasting serum glucose level and FINS was fasting serum insulin level.

### 2.8. Histological Examination [18]

Liver and pancreas samples were fixed with 10% formalin, stained with hematoxylin and eosin (H&E), and then observed under an Olympus microscope BX53 equipped with a CCD camera (Olympus, Tokyo, Japan) using the DP2-BSW image analysis software system (Olympus, Tokyo, Japan) at the 400-fold magnification.

### 2.9. Statistical Analysis

Data were expressed as mean ± standard deviation of the number of animals (*n* = 8) used in each experiment. Statistical analysis was performed using Duncan's new multiple range test by software of DPS 7.5. Correlation analysis was performed by software of PASW Statistics 18, and significance was analyzed by test of two-tailed. Significance was accepted at *P* < 0.05. Origin 9.0 software was used for plotting graphs.

## 3. Results

### 3.1. Effects of COE on Changes in FBG with Time

FBG was determined weekly at the initiation of treatment (W0), one week after treatment (W1), two weeks after treatment (W2), three weeks after treatment (W3), and four weeks after treatment (W4); the results were listed in Table 2. On W0, FBG was significantly higher in group DM as compared to that in group NC (*P* < 0.01). After one week of treatment, FBG in group COE-L, COE-H, and ROS was significantly decreased, but group DM still stayed at an initial high level. However, the treatment seemly lost its effectiveness over time; FBG in W2, W3, and W4 became higher than that in W1, which was a strange phenomenon. Several causes to this phenomenon could be considered: during the first week of treatment, both medications and self-healing system reacted on diabetic model mice, so FBG in the treatment group diabetic model mice decreased dramatically and FBG in group DM mice presented little change; self-healing system in diabetic model mice destroyed by STZ became more and more serious over time; only medications played a major role in controlling FBG, which eventually lead to the FBG increasing in diabetic model mice after two weeks' treatment. In fact, the treatment is beneficial to control the rise of FBG in another aspect. FBG was decreased significantly in other groups (*P* < 0.01) as compared to that in group DM at the same week. In general, FBG in group COE-L, COE-H, and ROS declined at the end of the treatment in varying degrees while group DM showed more serious hyperglycemia.

### 3.2. Effects of COE on OGTT

**Figure 1:**
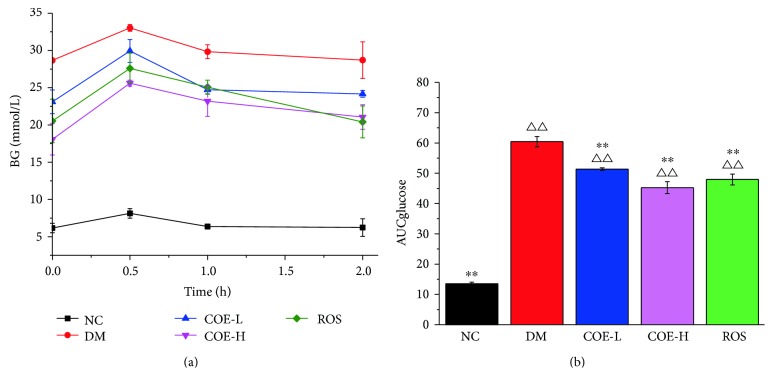
Effects of COE on oral glucose tolerance test (OGTT) (a) and area under curve (AUC) (b) in diabetic model mice. BG: blood glucose level. AUCglucose represented area under curve was calculated from blood glucose level. Mice were supplemented with COE at 50 (COE-L) or 200 (COE-H) mg/kg body weight and rosiglitazone at 4 mg/kg body weight (ROS) for 3 weeks. Data were presented as mean ± standard deviation (*n* = 3). ^∗∗^*P* < 0.01 compared to group DM; ^ΔΔ^*P* < 0.01 compared to group NC.

The oral glucose tolerance test was performed as section 2.6 described. Blood samples were collected from the tail vein of mice and analyzed for glucose content at 0 , 0.5 h, 1 h, and 2 h. The blood glucose level was significantly higher in group DM than in group NC, COE-H, and ROS at different temporal points as shown in Table 3. It is obvious that serious impaired glucose tolerance appeared in group DM, and other treatment groups had some improvement. After intragastric administration of glucose for 0.5 h, blood glucose levels of all mice were up to the peak and then went down in different range (Figure 1(a)). Except group NC, COE-H-treated mice in OGTT showed the lowest AUC value with reduction of 25.1% compared with group DM (Figure 1(b)). Namely, group COE-H showed greater glucose tolerance compared with other treatment groups.

### 3.3. Effects of COE on Insulin Sensitivity

**Figure 2:**
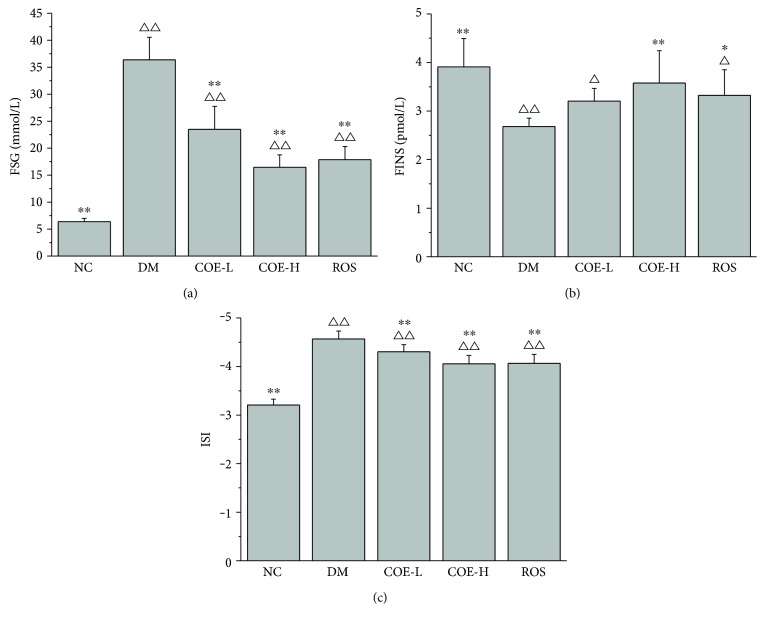
Effects of COE on FSG (a), FINS (b), and ISI (c) in diabetic model mice. FSG: fast serum glucose level; FINS: fast serum insulin level; ISI: insulin sensitivity index. Mice were supplemented with COE at 50 (COE-L) or 200 (COE-H) mg/kg body weight and rosiglitazone at 4 mg/kg body weight (ROS) for 4 weeks. Data were presented as mean ± standard deviation (*n* = 8). ^∗^*P* < 0.05 and ^∗∗^*P* < 0.01 compared to group DM; ^Δ^*P* < 0.05 and ^ΔΔ^*P* < 0.01 compared to group NC.

The serum samples were withdrawn after the animals being sacrificed for determination of glucose content and insulin level. As shown in Figure 2(a), a significant reduction in fasting glucose serum levels of COE and rosiglitazone treated diabetic model mice, compared with group DM (*P* < 0.01). Group DM showed a lower insulin level as compared to group NC (*P* < 0.01); insulin level in group COE-H was lower than in group NC, but there was no significant difference between them; group COE-L showed no significant difference as compared to group DM; a significant augment in insulin level was observed in group ROS as compared to group DM (*P* < 0.05), whereas it was significantly lower than group NC (*P* < 0.05) (Figure 2(b)). In the case of treatment groups, a significant increase in ISI was observed as compared to group DM (*P* < 0.01) (Figure 2(c)).

### 3.4. Effects of COE on Lipid Profile

After 4 weeks of treatment, serum lipid levels of group COE-L, COE-H, and ROS were improved on the whole (Table 4). Data revealed that TG, TC, and LDLC in group DM were significantly higher than in other groups and HDLC was lower than them. Concrete changes of each serum lipid parameter were displayed as follows: TG in group COE-L, COE-H, and ROS was reduced compared with that in group DM by 20.4%, 34.5%, and 35.0%, respectively; TC was reduced by 26.6%, 28.1%, and 34.6% corresponding to group COE-L, COE-H, and ROS, respectively; high dose of COE treatment resulted in significant increase of HDLC as compared to group DM, which was an approach to the levels of HDLC in the normal healthy mice; all treatment groups showed significantly (*P* < 0.01) lower LDLC when compared with group DM. Obviously, high dose of COE treatment had the best effect on improvement of serum lipid levels in diabetic model mice.

### 3.5. Antioxidant Analysis in Serum and Liver

**Figure 3:**
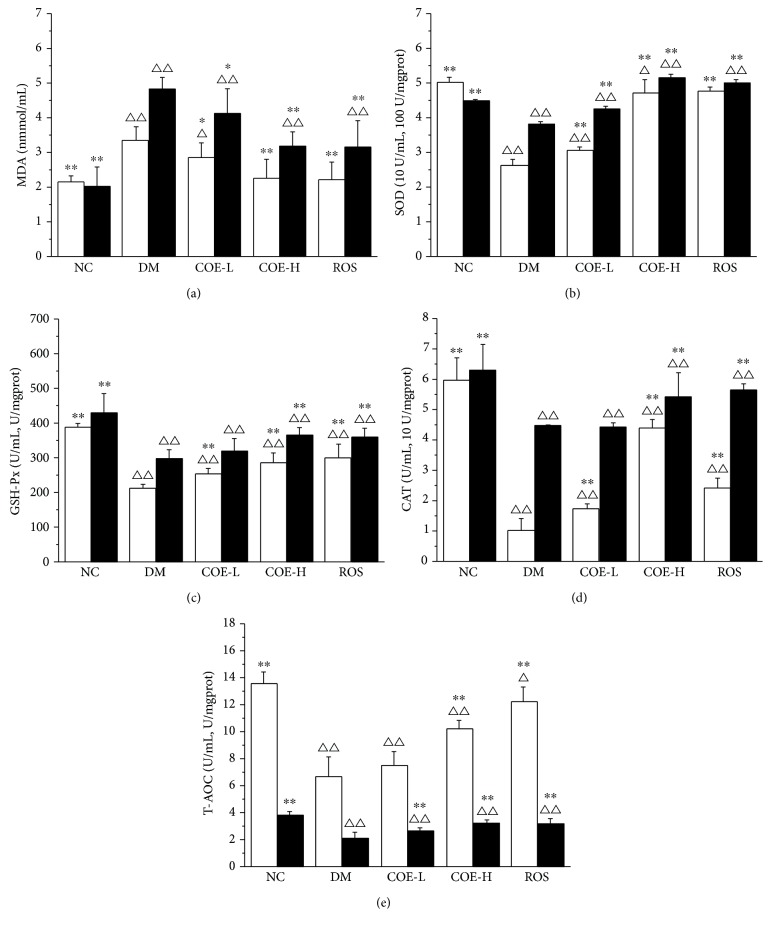
Effects of COE on MDA (a), SOD (b), GSH-Px (c), CAT (d), and T-AOC (e) in the serum (□) and liver (■) in diabetic model mice. MDA: the content of malondialdehyde; T-AOC: total antioxidant capacity; SOD: activity of superoxide dismutase; GSH-Px: activity of glutathione peroxidase; CAT: activity of catalase. Mice were supplemented with COE at 50 (COE-L) or 200 (COE-H) mg/kg body weight and rosiglitazone at 4 mg/kg body weight (ROS) for 4 weeks. In the y-coordinate of graphs (b), (c), (d), and (e), where two units are present, one unit was U/mL or linked with U/mL represented the unit of serum indexes and another unit was U/mgprot or linked with U/mgprot represented the unit of liver indexes. Data were presented as mean ± standard deviation (*n* = 8). ^∗^*P* < 0.05 and ^∗∗^*P* < 0.01 compared to group DM; ^Δ^*P* < 0.05 and ^ΔΔ^*P* < 0.01 compared to group NC.

Antioxidant analysis was performed on the determining the content of MDA, activities of enzymatic antioxidants (SOD, GSH-Px, and CAT), and T-AOC in the serum and liver of each group. The content of MDA was lower in each treatment group either in serum or liver compared with that in group DM; high dose of COE treatment and rosiglitazone treatment showed a significant (*P* < 0.01) decrease by 32.5% and 33.7% in serum and 34.2% and 34.8% in liver, respectively (Figure 3(a)). After 4 weeks of supplementing diabetic model mice with COE, activities of SOD, GSH-Px, and CAT in serum and liver were all enhanced in a dose-dependent manner (Figures 3(b), 3(c), and 3(d)). Furthermore, COE supplementation especially in high dose restored the levels of serum and liver T-AOC. From an overall perspective, COE could improve antioxidant activity of diabetic model mice, especially high dose of it exhibited the remarkable effect, but there was still a great difference between the antioxidant activity of COE treatment diabetic model mice and normal mice.

### 3.6. Correlation Analysis between Glycolipid Metabolism and Antioxidant Capacity in COE-Treated Diabetic Model Mice

In order to evaluate whether there is a relationship between glycolipid metabolism and antioxidant capacity after diabetic model mice were treated by COE, we analyzed the correlation between glycolipid metabolism indexes (FSG, ISI, TG, TC, HDLC, and LDLC) and antioxidant indexes (MDA, SOD, GSH-Px, CAT, and T-AOC) in serum. The result of correlation analysis was showed in Table 5. Significant correlation could be observed between FSG and 5 kinds of antioxidant indexes from Table 5. In COE-treated diabetic model mice, FSG was positively associated with SOD, GSH-Px, CAT, and T-AOC (*P* < 0.05, *P* < 0.05, *P* < 0.01, and *P* < 0.05) and was negatively associated with MDA (*P* < 0.05). The correlation between INS and MDA was significant at 0.01 level. ISI was correlated with MDA, SOD, GSH-Px, CAT, and T-AOC but was only significantly correlated with GSH-Px and CAT at 0.05 level. Weaker associations were present between TG and antioxidant indexes or TC and antioxidant indexes, as well as no significant difference. There were significant positive correlations between HDLC and SOD, GSH-Px, CAT, and T-AOC at the level of 0.05, 0.05, 0.01, and 0.05, respectively. It was also showed that LDLC was strongly correlated with antioxidant indexes but was only significantly correlated with MDA (*P* < 0.01) and T-AOC (*P* < 0.05). On the whole, different degrees of correlation between glycolipid metabolism and antioxidant capacity existed in COE-treated diabetic model mice.

### 3.7. Effects of COE on Liver Function

The change of liver coefficient (LC) and the content of transaminase and alkaline phosphatase in serum were intuitive evidences which could reflect liver damage. As shown in Table 6, the LC and serum ALT, AST, and ALP levels in group DM mice significantly increased as compared with those in group NC mice (*P* < 0.01). After treatment with COE, liver function of diabetic model mice was improved as follows: the LC in group COE-H was remarkably reduced and was near to normal level; serum ALT, AST, and ALP levels in high-dose COE-treated diabetic model mice were all lower than in diabetic model mice with 67.0%, 36.2%, and 52.4% reduction, respectively. Although each index except ALT in low dose of COE-treated diabetic model mice was reduced in comparison to diabetic model mice, there was no significant difference between them.

### 3.8. Effects of COE on Morphological Changes of Pancreas and Liver

**Figure 4:**
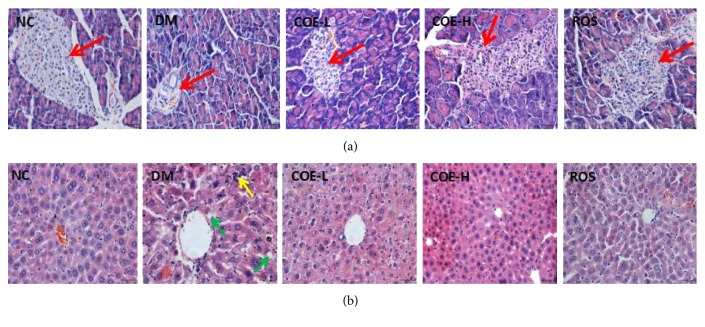
Hematoxylin-eosin staining of the pancreas (a) and liver (b) in each group. Mice were supplemented with COE at 50 (COE-L) or 200 (COE-H) mg/kg body weight and rosiglitazone at 4 mg/kg body weight (ROS) for 4 weeks. Where, the red arrow pointed was pancreas islet, the green arrow pointed was fat droplet, and the yellow arrow pointed was inflammatory cells. The black English word on each picture represented the pictures' corresponding group.

Figure 4 illustrated representative photographs of 0.5 *μ*m thin slice of pancreas and liver stained with H&E. Group NC mice showed clear margin in pancreas islet with normal cellular population of islet cells being regularly arranged and evenly distributed. Group DM mice showed severe atrophies in pancreas islet and remarkable decrease in the number of islet cells. Low-dose and high-dose supplementation of COE exerted a protective effect against the damage induced by STZ, which were mainly reflected in the significant increase of cellular population of islet. Rosiglitazone-treated mice were observed with some improvement in pancreas islet as nearly the same as that of COE-H-treated mice. In Figure 2(b), group NC mice showed intact cellularity, compact arrangement, clear cell boundaries of hepatocytes, and no invisible fat droplets in pathological section of liver. Group DM mice showed that lots of spherical vacuoles of fat droplets were accumulated in hepatocytes. In addition, histomorphological features of liver in group DM appeared with irregular arrangement, obscure boundary, and serious swell of hepatocytes and infiltration of inflammatory cells. Liver impairment in each treatment group greatly improved as compared to that in group DM mice.

## 4. Discussion

In the present study, we investigated effects of 4 weeks of COE treatment on the glycolipid metabolism and resistance to oxidative stress in diabetic model mice induced by combining feeding high-glucose-fat diet with intraperitoneal injection of streptozotocin. Serious damage of beta-islet cells induced by STZ triggered insufficient insulin secretion and resulted in excessive amounts of glucose in the blood [19]. High-glucose-fat diet induced insulin resistant with decreasing glucose tolerance and insulin sensitivity [20]. The method of establishing diabetic model mice was less expensive, easily available, and relatively shorter period consuming for development [21]. Rosiglitazone is an antidiabetic drug of thiazolidinedione, which can effectively control blood glucose mainly by increasing the insulin sensitivity.

Four weeks of oral administration of COE made significant improvement in the FBG or FSG of diabetic model mice, as well as rosiglitazone did. High dose of COE decreased FBG or FSG more efficiently than low dose of COE, and rosiglitazone exhibited nearly the same effect on decreasing FBG or FSG. In addition, we observed that high dose of COE and rosiglitazone treatment resulted in a notable increase in the depressed serum insulin concentration and insulin sensitivity in diabetic model mice; high dose of COE significantly improved serum insulin concentration better than rosiglitazone, but low dose of COE just increased insulin sensitivity more significantly. All treatments improved glucose tolerance, especially for high dose of COE treatment. The above results illustrated that COE decreasing FBG or FSG might be due to its protection to insulin secretion of beta-islet cells, as well as reduction of insulin resistance; rosiglitazone exerted the glucose-lowering effect mainly due to reduction of insulin resistance. Increasing insulin production and reducing insulin resistance are significant for the treatment of diabetes [22]. Therefore, COE was better for regulating glucose metabolism than rosiglitazone but was dose-dependent.

The results of serum lipid profile revealed significant amelioration of dyslipidemia in COE- and rosiglitazone-treated diabetic model mice. Among these three treatment groups, group COE-H mice presented nearly normal level in each lipid index, group ROS mice improved well but were little inferior to group COE-H mice, and group COE-L mice presented the effect of improvement for the last one. The abnormality of lipid metabolism often occurring with diabetes is a major risk factor for diabetic complications especially for some cardiovascular diseases [23]. Furthermore, abnormal lipid metabolism interacts with insulin resistance and improving lipid metabolism disorder is important for preventing and controlling diabetes [24]. High-glucose-fat diet could induce mice to possess lipid metabolism abnormality which was characterized by high TG levels. Then it would further develop insulin resistance by different mechanisms but considered mainly through Randle or glucose-fatty acid cycle [25]. Insulin is considered as the main factor in the inhibition of lipolysis [26]. In the presence of insulin resistance, the use of glucose in peripheral tissues has been obstructed, and insulin cannot effectively suppress lipolysis, so that superfluous free fatty acids are released. However, excessive free fatty acids cannot be sufficiently used by muscles and adipose tissue and become ingredients for liver producing more TG, TC, and LDLC and less HDLC [27]. The observation of improvement effect on serum lipids by rosiglitazone was depending on that rosiglitazone-activated PPAR alpha and therefore affected relevant apolipoprotein [28]. From our results, it was expected that one of the reasons for why COE exhibited effect on improving serum lipid levels in diabetic model mice may be increase in both insulin concentrations and peripheral tissue sensibility to insulin. Obviously, high dose of COE remarkably normalized serum lipid levels; it might alter serum lipid directly by influencing lipoprotein synthesis and/or catabolism.

In our study, the levels of SOD, GSH-Px, CAT, MDA, and T-AOC in the serum and liver were tested to assess the effect of COE on antioxidant capacity in diabetic model mice. The results revealed that activity of each kind of antioxidase activity was enhanced, the content of MDA was reduced under the 4 weeks of intervention of COE, as well as T-AOC level was improved. High dose of COE was better than low dose about impacting on antioxidant capacity in diabetic model mice which suggested dose-dependent effect of COE. The effect of rosiglitazone on antioxidant capacity in diabetic model mice was even close to that of high dose of COE. Free radical reaction plays an important role in the body's defense mechanisms. Under normal circumstances, the generation of free radicals keeps a dynamic balance with elimination of free radicals. But long-term high blood glucose level will lead to generating reactive oxygen species and thus trigger a series of free radical chain reactions to generate more free radicals [29]. Antioxidase system including SOD, GSH-Px and, CAT makes a great contribution to get rid of overproduced free radicals; they are even in effective resistance to glucose toxicity, thus delaying or preventing the pancreatic cells being damaged [30]. The content of MDA is often measured to show the degree of lipid peroxidation which can cause gene mutations, make the abnormal protein expression, and become the important factor of a variety of chronic diseases such as pancreatic injury in diabetes [31, 32]. Several studies reported rosiglitazone improved vivo antioxidant [33, 34]; similar result was obtained in the present study. It is expected that COE enhanced the antioxidant capacity in diabetic model mice thereby preventing pancreas islet from being further impaired.  Figure 4(a) showed the histopathological improvement of pancreas islet in diabetic model mice under the treatment of COE and rosiglitazone.

The results of correlation analysis indicated that FSG was inversely correlated to activity of antioxidant enzymes and positively correlated to content of lipid peroxide MDA in the COE-treated diabetic model mice. Other indexes of glycolipid metabolism except FSG showed varying degrees of correlation relationship with different antioxidant indexes, but not all significant. These correlations suggested COE may enhance antioxidant capacity of treated diabetic model mice by decreasing the glucose toxicity to tissues. Lipid regulation by COE was more likely to depend on other mechanism, such as influencing lipoprotein synthesis and/or catabolism.

We tested liver function to evaluate whether COE presented protective or adverse effect. The enzyme activities including AST, ALT, and ALP in serum could sensitively reflect the extent and type of hepatocyte damage [35, 36]. Under normal conditions, the vast majority of AST, ALT, and ALP resides in hepatocytes. When hepatocytes are damaged, these enzymes will be released into blood which results in their higher levels in serum [37]. Not only increase in LC and activities of AST, ALT, and ALP but also morphological changes showed severe liver damage in combination high-glucose-fat diet with STZ-induced diabetic model mice. But surprisingly, oral administration of COE was conducive to restore liver function of diabetic model mice, which was demonstrated by the remarkable decrease in liver function indexes including LC, AST, ALT, and ALP levels compared to diabetic model mice. Improvement of liver function indexes simultaneously indicated COE did not have obvious adverse effects. However, high dose of COE was prior to low dose of COE and rosiglitazone in regards of liver protection. Rosiglitazone protected diabetic rats from liver destruction by decreasing hepatocyte apoptosis and downregulating the expression levels for hepatic cyclooxygenase-2 (COX-2) mRNA, Fas ligand (FasL) mRNA, and COX-2 protein [38]. It needs further investigation to explore whether the mechanism of COE for liver protection is same as that of rosiglitazone. But the presence of impact on liver protection by COE did not negate the fact that COE played an important role in enhancing antioxidation which was related with reduction of liver damage.

From the perspective of the whole results of the present study, we summarized the mechanism for COE effecting on glycolipid metabolism and antioxidant capacity in diabetic model mice. On the one hand, COE improved antioxidant capacity of diabetic model mice and lead to restoration of function of beta-islet cells, then insulin was released more to blood, and blood glucose was disposed by the insulin signaling pathway. Glucose toxicity to tissues was alleviated to do less harm to antioxidase system, and antioxidant capacity of diabetic model mice was improved in turn. On the other hand, high-glucose-fat diet caused not only dyslipidemia but also insulin resistance; COE might regulate serum lipid level by protecting liver function to perform normal lipid synthesis and catabolism; therefore, insulin resistance was also improved. In our previous study, constituent analysis of COE was preformed and we found rutin, hyperin, isoquercitrin, luteoloside, astragalin, quercetin, naringenin, and kaempferol were mainly flavonoid compounds in COE [16]. Flavonoids have a wide range of physiological activities; they can not only reduce blood glucose level and regulate lipid metabolism but also possess antioxidant properties [39–41]. The activities of COE shown in our study might be closely associated with those flavonoids. Further studies are needed to elucidate the exact ingredients in COE which could improve physiological function of diabetic model mice.

## 5. Conclusion

In conclusion, COE exhibited significant ameliorative effects on regulating glucose and lipid metabolism in high-glucose-fat diet-fed and streptozotocin-induced diabetic model mice. Pancreas islets and liver of COE-treated diabetic model mice showed some recovery in histological findings. The capacity to resist oxidative stress in COE-treated diabetic model mice was enhanced notably which could explain why pancreas islets and liver showed improvement in morphology and function. Furthermore, glucose metabolism had positive correlation between antioxidant capacities, but lipid metabolism did not have obvious correlation relationship with antioxidant capacity in COE-treated mice. COE improved the levels of various biochemical indexes by dose-dependent. All results indicated that COE was expected to be used as a supplement to treat or prevent diabetes. However, further investigations about signal pathway related with glycolipid metabolism and oxidative stress in diabetic model mice are appreciated to carry out.

## Figures and Tables

**Table 1:** List of animal groups with different treatments.

Groups	Status and treatment
NC	Healthy, treated with 0.5% CMC-Na solution in equivalent volume to the test treatments
DM	Diabetic, treated with 0.5% CMC-Na solution in equivalent volume to the test treatments
COE-L	Diabetic, treated with low dose of *Chimonanthus nitens* Oliv. leaf extract (50 mg/kg body weight)
COE-H	Diabetic, treated with high dose of *Chimonanthus nitens* Oliv. leaf extract (200 mg/kg body weight)
ROS	Diabetic, treated with rosiglitazone (4 mg/kg body weight)

NC was normal control group, DM was diabetic model group, COE-L was low dose of *Chimonanthus nitens* Oliv. leaf extract group, COE-H was high dose of *Chimonanthus nitens* Oliv. leaf extract group, and ROS was rosiglitazone group. There were 8 animals in each group. Treatments were started after three days of streptozotocin injection.

**Table 2:** Effects of COE on FBG in diabetic model mice with time.

Groups	FBG (mmol/L)				
	W0	W1	W2	W3	W4
NC	6.20 ± 0.19^∗∗^	6.60 ± 0.25^∗∗^	7.05 ± 0.86^∗∗^^#^	6.21 ± 0.71^∗∗^	6.44 ± 0.58^∗∗^
DM	22.03 ± 2.78^ΔΔ^	22.30 ± 2.12^ΔΔ^	26.71 ± 2.20^ΔΔ##^	28.73 ± 2.92^ΔΔ##^	27.71 ± 2.74^ΔΔ##^
COE-L	22.28 ± 4.20^ΔΔ^	17.16 ± 2.45^∗∗^^ΔΔ#^	17.99 ± 1.44^∗∗^^ΔΔ#^	23.09 ± 1.43^∗∗^^ΔΔ^	20.61 ± 4.05^∗∗^^ΔΔ^
COE-H	21.46 ± 5.22^ΔΔ^	15.36 ± 3.92^∗∗^^ΔΔ##^	14.74 ± 1.23^∗∗^^ΔΔ##^	18.31 ± 2.81^∗∗^^ΔΔ^	17.18 ± 2.78^∗∗^^ΔΔ^
ROS	21.01 ± 2.89^ΔΔ^	16.08 ± 3.90^∗∗^^ΔΔ##^	17.71 ± 3.42^∗∗^^ΔΔ^	20.46 ± 2.78^∗∗^^ΔΔ^	17.74 ± 2.34^∗∗^^ΔΔ^

Data were presented as mean ± standard deviation (*n* = 8). FBG: fast blood glucose level. FBG of each group was determined weekly at the initiation of treatment (W0), one week after treatment (W1), two weeks after treatment (W2), three weeks after treatment (W3), and four weeks after treatment (W4). ^∗∗^*P* < 0.01 compared to group DM; ^ΔΔ^*P* < 0.01 compared to group NC; ^#^*P* < 0.05 and ^##^*P* < 0.01 compared to W0.

**Table 3:** Effects of COE on OGTT in diabetic model mice.

Groups	BG (mmol/L)			
	0 h	0.5 h	1 h	2 h
NC	6.17 ± 0.64^∗∗^	8.13 ± 0.64^∗∗^	6.37 ± 0.32^∗∗^	6.23 ± 1.17^∗∗^
DM	28.67 ± 0.25^ΔΔ^	33.03 ± 0.46^ΔΔ^	29.83 ± 0.93^ΔΔ^	28.70 ± 2.46^ΔΔ^
COE-L	23.10 ± 1.59^∗∗^^ΔΔ^	29.93 ± 1.53^ΔΔ^	24.73 ± 0.55^∗^^ΔΔ^	24.17 ± 0.45^∗∗^^ΔΔ^
COE-H	18.07 ± 2.10^∗∗^^ΔΔ^	25.60 ± 0.40^∗∗^^ΔΔ^	23.20 ± 2.05^∗∗^^ΔΔ^	21.07 ± 1.65^∗∗^^ΔΔ^
ROS	20.53 ± 2.91^∗∗^^ΔΔ^	27.60 ± 2.04^∗∗^^ΔΔ^	25.07 ± 0.95^∗^^ΔΔ^	20.40 ± 2.14^∗∗^^ΔΔ^

Data were presented as mean ± standard deviation (*n* = 3). OGTT: oral glucose tolerance test; BG: blood glucose level. After three weeks of treatment, all animals were intragastric administrated with 2.0 g/kg body wt. of glucose and then their blood glucose level were measured at 0, 0.5 h, 1 h, and 2 h. ^∗^*P* < 0.05 and ^∗∗^*P* < 0.01 compared to group DM; ^ΔΔ^*P* < 0.01 compared to group NC.

**Table 4:** Effects of COE on serum lipid profile in diabetic model mice after 4 weeks of treatment.

Groups	TG (mmol/L)	TC (mmol/L)	HDLC (mmol/L)	LDLC (mmol/L)
NC	1.03 ± 0.38^∗∗^	1.81 ± 0.41^∗∗^	2.34 ± 0.24^∗∗^	0.20 ± 0.05^∗∗^
DM	2.06 ± 0.43^ΔΔ^	3.27 ± 0.23^ΔΔ^	1.28 ± 0.36^ΔΔ^	0.44 ± 0.09^ΔΔ^
COE-L	1.64 ± 0.23^∗∗^^Δ^	2.40 ± 0.47^∗∗^	1.52 ± 0.37^ΔΔ^	0.32 ± 0.09^∗∗^^ΔΔ^
COE-H	1.35 ± 0.23^∗∗^	2.35 ± 0.52^∗∗^	2.17 ± 0.28^∗∗^	0.23 ± 0.06^∗∗^
ROS	1.34 ± 0.53^∗∗^	2.14 ± 0.90^∗∗^	2.09 ± 0.45^∗∗^^Δ^	0.27 ± 0.06^∗∗^

Data were presented as mean ± standard deviation (*n* = 8). TG: triglyceride; TC: total cholesterol; HDLC: high-density lipoprotein cholesterol; LDLC: low-density lipoprotein cholesterol. Mice were supplemented with COE at 50 (COE-L) or 200 (COE-H) mg/kg body weight and rosiglitazone at 4 mg/kg body weight (ROS) for 4 weeks. ^∗∗^*P* < 0.01 compared to group DM; ^Δ^*P* < 0.05 and ^ΔΔ^*P* < 0.01 compared to group NC.

**Table 5:** Correlation between glycolipid metabolism and antioxidant capacity in COE-treated diabetic model mice.

BI		FSG	INS	ISI	TG	TC	HDLC	LDLC
MDA	*r*	0.650^∗^	−0.816^∗∗^	−0.267	0.399	0.099	−0.266	0.845^∗∗^
	*P*	0.022	0.001	0.401	0.198	0.759	0.402	0.001
SOD	*r*	−0.610^∗^	0.251	0.533	−0.409	0.068	0.669^∗^	−0.571
	*P*	0.035	0.431	0.074	0.187	0.833	0.017	0.052
GSH-Px	*r*	−0.695^∗^	0.438	0.612^∗^	−0.449	−0.183	0.663^∗^	−0.554
	*P*	0.012	0.155	0.034	0.143	0.569	0.019	0.062
CAT	*r*	−0.737^∗∗^	0.308	0.665^∗^	−0.514	−0.117	0.744^∗∗^	−0.574
	*P*	0.006	0.330	0.018	0.087	0.717	0.006	0.051
T-AOC	*r*	−0.687^∗^	0.391	0.548	−0.316	−0.008	0.590^∗^	−0.681^∗^
	*P*	0.014	0.209	0.065	0.317	0.980	0.043	0.015

BI: biochemical index; *r*: Pearson's correlation coefficient; *P*: significant difference value; ^∗^correlation was significant at the 0.05 level (2-tailed); ^∗∗^correlation was significant at the 0.01 level (2-tailed), *n* = 12.

**Table 6:** The LC and serum ALT, AST, and ALP levels in each group after 4 weeks of treatment.

Groups	LC (mg/g)	ALT (U/L)	AST (U/L)	ALP (U/L)
NC	46.47 ± 3.80^∗∗^	24.14 ± 7.22^∗∗^	66.29 ± 6.65^∗∗^	37.43 ± 9.57^∗∗^
DM	57.40 ± 8.21^ΔΔ^	151.43 ± 27.23^∗∗^^ΔΔ^	213.86 ± 22.36^ΔΔ^	141.86 ± 35.04^ΔΔ^
COE-L	54.55 ± 3.73^Δ^	99.67 ± 18.11^∗∗^^ΔΔ^	187.00 ± 53.74^ΔΔ^	130.00 ± 58.61^ΔΔ^
COE-H	49.86 ± 2.70^∗^	50.00 ± 19.13^∗∗^^Δ^	136.50 ± 62.86^∗^^Δ^	67.50 ± 13.85^∗∗^
ROS	50.68 ± 7.79^∗^	65.00 ± 17.25^∗∗^^ΔΔ^	142.33 ± 56.45^∗^^Δ^	95.50 ± 17.65^∗^^ΔΔ^

Data were presented as mean ± standard deviation (*n* = 8). LC: liver coefficient; ALT: glutamic-pyruvic transaminase; AST: aspartate aminotransferase; ALP: alkaline phosphatase. Mice were supplemented with COE at 50 (COE-L) or 200 (COE-H) mg/kg body weight and rosiglitazone at 4 mg/kg body weight (ROS) for 4 weeks. ^∗^*P* < 0.05 and ^∗∗^*P* < 0.01 compared to group DM; ^Δ^*P* < 0.05 and ^ΔΔ^*P* < 0.01 compared to group NC.
